# Transcriptomic analysis of the venom gland of the red-headed krait (*Bungarus flaviceps*) using expressed sequence tags

**DOI:** 10.1186/1471-2199-11-24

**Published:** 2010-03-29

**Authors:** Ang Swee Siang, Robin Doley, Freek J Vonk, R Manjunatha Kini

**Affiliations:** 1 Department of Biological Sciences, National University of Singapore, 10 Kent Ridge Road, Singapore 117546, Singapore; 2 Department of Molecular Biology and Biotechnology, Tezpur University, Tezpur-784 028, Assam, India; 3 Institute of Biology, Leiden University, Sylvius Laboratory, Sylviusweg 72, 2333 BE, Leiden, The Netherlands; 4 Department of Biochemistry and Molecular Biology, Medical College of Virginia, Virginia Commonwealth University, Richmond, Virginia 23298-0614, USA

## Abstract

**Background:**

The Red-headed krait (*Bungarus flaviceps*, Squamata: Serpentes: Elapidae) is a medically important venomous snake that inhabits South-East Asia. Although the venoms of most species of the snake genus *Bungarus *have been well characterized, a detailed compositional analysis of *B. flaviceps *is currently lacking.

**Results:**

Here, we have sequenced 845 expressed sequence tags (ESTs) from the venom gland of a *B. flaviceps*. Of the transcripts, 74.8% were putative toxins; 20.6% were cellular; and 4.6% were unknown. The main venom protein families identified were three-finger toxins (3FTxs), Kunitz-type serine protease inhibitors (including chain B of β-bungarotoxin), phospholipase A_2 _(including chain A of β-bungarotoxin), natriuretic peptide (NP), CRISPs, and C-type lectin.

**Conclusion:**

The 3FTxs were found to be the major component of the venom (39%). We found eight groups of unique 3FTxs and most of them were different from the well-characterized 3FTxs. We found three groups of Kunitz-type serine protease inhibitors (SPIs); one group was comparable to the classical SPIs and the other two groups to chain B of β-bungarotoxins (with or without the extra cysteine) based on sequence identity. The latter group may be functional equivalents of dendrotoxins in *Bungarus *venoms. The natriuretic peptide (NP) found is the first NP for any Asian elapid, and distantly related to Australian elapid NPs. Our study identifies several unique toxins in *B. flaviceps *venom, which may help in understanding the evolution of venom toxins and the pathophysiological symptoms induced after envenomation.

## Background

Snake venom is a complex mixture of biologically active proteins and peptides that exert very powerful and specific effects. This mixture is interesting from the angle of molecular evolution, as the genes encoding the venom ingredients seem to undergo some form of hypermutation resulting in accelerated evolution and a staggering diversity of isoforms [1,2], sometimes functionally and structurally radically different. The basis for this phenomenon seems to be due to gene-duplication and diversification of existing venom genes. This results in a highly dynamic venom composition both at the interspecific and intraspecific level [3]. This allows the snake to deal with a wide array of different prey items. Snake venom is also a valuable resource for proteins and peptides that may serve as lead compounds to treat certain human disorders [4]. Examining the transcriptome of a venom gland will also reveal venom proteins that are low abundant, which is crucial to both expanding the resource of pharmaceutical compounds as well as to understand the evolution of snake venom proteins [5]. Further, cataloguing of snake venom proteins through transcriptomic analysis may help to understand the pathophysiological symptoms induced after envenomation and correlates with the venom composition [6-12].

For example, we have recently used transcriptomic analysis to show the presence of three-finger toxins (3FTxs) in viperid venom [13,14]. By elucidating the gene structures of these toxins we could infer their relationship with the elapid 3FTxs, which helped us to understand the evolution of this toxin protein family. Hence, snake venom gland transcriptomes continue to be a valuable tool in improving our understanding of snake venom composition and evolution, management of snake bite, and the opportunity to identify and study the function of the low abundant proteins.

Kraits (*Bungarus *species) belong to the family Elapidae. They are one of the better studied snakes of the world. They are widely distributed across South and Southeast Asia and are highly venomous [15]. Many biologically important proteins, particularly α- bungarotoxins, κ- bungarotoxins and β- bungarotoxins, have been well characterized from the venom of *Bungarus *species. The first two belong to the 3FTx family, whereas the last one is a covalent heterodimer of phospholipase A_2 _(PLA_2_) and a serine protease inhibitor (SPI) -like polypeptide [16-18]. α- bungarotoxin is a highly specific toxin that binds to peripheral nicotinic acetylcholine receptors (nAChRs) and it played a key role in the isolation and characterization of mammalian nAChRs [19]. Similar to other long-chain neurotoxins, it also binds to neuronal α7 nAChRs [20]. κ- bungarotoxins specifically bind to neuronal nAChRs (α3β2, α4β2 and α3β4) [20]. On the other hand, β- bungarotoxins - the major lethal factors bind to voltage-sensitive potassium channels in the presynaptic site [21,22].

The *B. flaviceps*, commonly known as the Red-headed krait, has phenotypically distinctive coloring of blue and black body, and the head, neck and tail are bright red in color. The venom of *B. flaviceps *is more potent than *B. fasciatus *but comparable in potency to *B. candidus *venom; the LD_50 _values of *B. flaviceps*, *B. candidus *and *B. fasciatus *venoms are 3.5 μg, 3.2 μg and 61.7 μg per kg of experimental mouse respectively [23]. Other than the isolation and characterization of β- bungarotoxin [15,24], κ- flavitoxin [25,26] and PLA_2 _[15], not much information is available on the venom of *B. flaviceps*. Therefore, we have here examined the venom gland of *B. flaviceps *by using expressed sequence tags (ESTs) to explore the venom composition in detail as well as to identify novel and low abundance toxins.

## Results and Discussion

### Composition of cDNA Library

**Figure 1:**
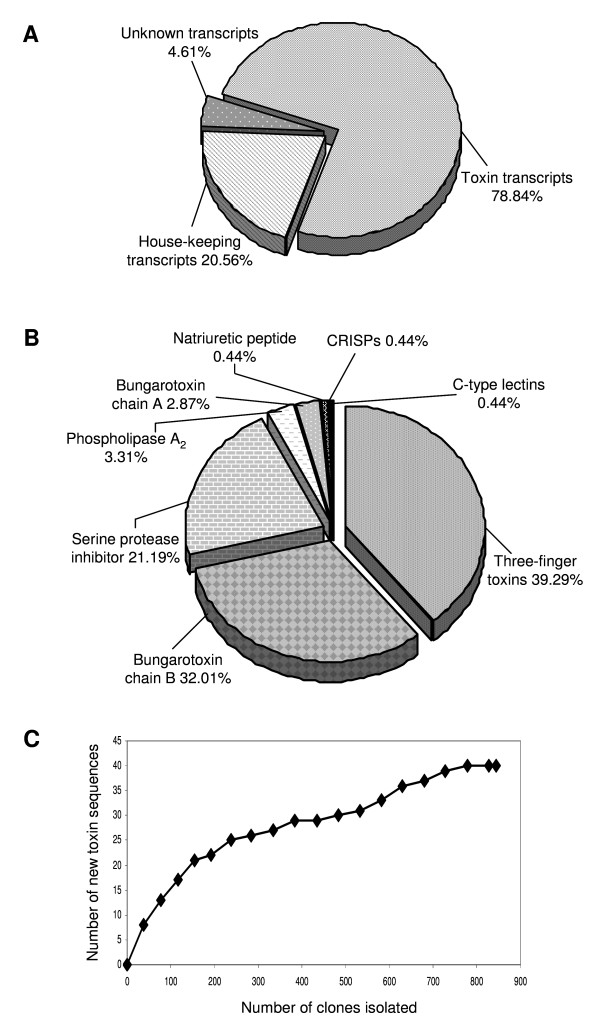
**Composition of a cDNA library from *B. flaviceps *venom gland tissue**. **A) **Relative abundance of genes in the cDNA library. **B) **Relative abundance of the toxin genes in the cDNA library. **C) **Graph showing the number of new toxins isolated reached a plateau when compared with the total number of sequences isolated.

We randomly selected 845 clones from the cDNA library and isolated the plasmids. 606 clones having inserts larger than 200 bp were numbered randomly and designated with BF (*Bungarus flaviceps*). Sequences were categorized based on the similarity shown in the analysis results and submitted to the database (Additional file 1). Accordingly, 74.84% of the ESTs were putative toxin transcripts, 4.61% were unknown transcripts and 20.56% of the ESTs were cellular transcripts (Figure 1A). Using sequence similarities to known toxins as a guide, all the putative toxin transcripts were further classified into different toxin families. Accordingly, the *B. flaviceps *ESTs contained transcripts encoding for six toxin families. Most clones encoded for 3FTxs (39.29, followed by β-bungarotoxin (Chains A and B) (34.88%), and Kunitz-type SPI (21.19%). The other toxin families are less well presented; clones encoding phospholipase A_2 _(PLA_2_) (6.26% including A chains of β-bungarotoxin), and two clones each of natriuretic peptide (NP), cysteine-rich secretory protein (CRISPs), and C-type lectin (Figure 1B). 3FTx is the major transcript of this venom gland library constituting 39.29% of the toxin transcripts. Similar observation was made in *Micrurus corallines *venom gland library which belongs to the elapid family where the 3FTx constitutes ~52% of the toxin transcripts [12]. This indicates that 3FTx is the major toxin of elapid snakes where as in viperid venoms proteases are the major venom components (Additional file 2). By comparing the total number of sequences *versus *number of new toxin sequences identified we showed that the number of new toxin transcripts was reaching an asymptote. Our results can thus be considered as a representative of the overall venom composition of *B. flaviceps *(Figure 1C). Although this study is not exhaustive, we have identified a number of low abundance transcripts (see below). We believe when the library contains high percentage of clones (~75%) coding for toxin-like proteins, sequencing 600-1000 clones may be sufficient to identify a number of low abundant clones.

### Three-Finger Toxin (3FTx) Family

Three-finger toxins are a group of low-molecular weight (<10 kDa), non-enzymatic polypeptides which have 60 to 74 amino acid residues [27]. They have 8-10 cysteine residues forming four or five disulphide bridges, of which four disulphide bridges are conserved [28]. Members of this family possess similar protein structures: three β- stranded loops extending from a central core, which is made up of the four highly conserved disulphide bridges [20,29], hence the name three-finger toxins. This 3FTxs family have been recruited in snake venom from a gene recruitment event within the SLUR/LYNX protein family [30], and their ancestral function is thought to be inhibition of the nicotinic acetylcholine receptor (nAChR) [30]. However, despite retaining the common three-finger motif, members of this family have since evolved a myriad of different functions. These include effects on: platelet function [31], different receptors associated with neurotransmission [32-38]; ion channels [39-41], viability of cardimyocytes [42] and red bloods cells [43], mitosis and apoptosis [44]; and effects on the cell membrane [45,46]. The different 3FTx members also vary considerably in binding affinity for the different receptors [47] and are important research ligands for studying receptors [48-50].

**Figure 2:**
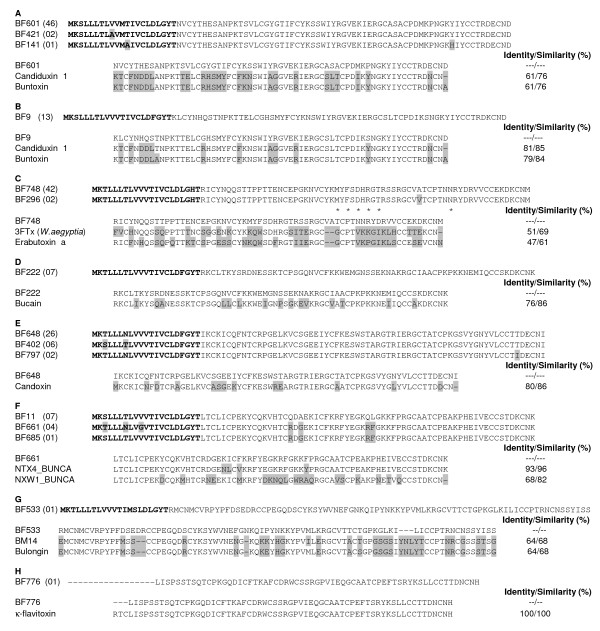
**Three-finger toxins (3FTxs) of *B. flaviceps *venom gland cDNA library**. Similar sequences were clustered together and a representative of the each cluster is presented. *B. flaviceps *3FTxs can be classified into eight major groups **A **through **H**. The deduced amino acid sequences of 3FTX isoforms of each group are shown. The amino residues which are different from the representative major isoform are shaded. The predicted signal peptide of the transcripts is shown bold. The major isoforms from each group is aligned with closely related 3FTx found in the database and the % identity and similarly are also shown. **(A and B) **Groups A and B are similar to candiduxin1 (Q8AY53.1) and buntoxin. Group B toxins are closer to candiduxin compared to group A toxins. **C) **Putative short-chain 3FTxs of *B. flaviceps *that are similar to 3FTx (ABX82864.1) from *W.aegyptia *and erabutoxin a (P60775.1). The residues of erabutoxin a that are involved in binding to nAChR receptor are shown in *. Critical functional residues in BF748 are replaced with Met, Phe and Asn (27th, 29th and 47th residues respectively). **D) **Group D toxins are similar to bucain (Q9YGI8.1). **E) **Group E non-conventional 3FTx and its alignment with candoxin (P81783.2). **F) **Group F non-conventional 3FTx and alignment with NTX4_BUNCA (Q6IZ95.1) and NXW1_BUNCA (Q8AY51.1). **G) **Group G non-conventional 3FTx and its alignment with BM14 (Q8JFX7.1) and bulongin. **H) **Group H 3FTx and its alignment with NXL2_BUNFL (κ-favitoxin) (P15815.1) (for details, see text).

Based on sequence similarities, 161 full length transcripts of 3FTxs were divided into 11 clusters and 4 singletons. These ESTs were segregated into eight distinct groups of toxins based on BLAST results (Figure 2). The first group has two clusters, BF601 and BF421 (46 and 2 clones) and a singleton (BF141); they all encode identical mature protein but have a single amino acid differences in the signal peptide regions and mature protein. They share 61% identity and 76% similarity to both candiduxin 1 and buntoxin (Kini RM, unpublished data) (Figure 2A). Similarly, BF9 (13 clones) encodes for a toxin that is also similar to candiduxin 1 from *B. candidus *(Orphan 3FTx IX subfamily; [51]) and buntoxin with 81% and 79% identity respectively (Figure 2B). Second cluster represented by clone BF9 show higher identity (81 and 79%) and similarity (85 and 84%) to the candiduxin1 and buntoxin than the above group of toxins. However the biological functions of candiduxin 1 and buntoxin are yet to be elucidated. The third group with two clusters, BF748 and BF296 (42 and 2 clones) show 51% identity to 3FTx III from *Walterinnesia aegyptia *[52] and 47% identity to erabutoxin a from *Laticauda semifasciata *(Figure 2C). In erabutoxin a Lys27, Trp29, Asp31, Arg33 and Lys47 are important in the binding to Torpedo nAChR [53,54]. Interestingly, the toxin encoded by BF748 has 27th, 29th and 47th residues replaced with Met, Phe and Asn respectively but the 31st and 33rd residues are conserved. Lower structural similarity combined with replacement of critical residues in the functional site may confer distinct pharmacological properties or specificities to these 3FTxs. One of the clusters, BF222 (7 clones) displays sequence similarity (76% identity; 86% similarity) (Figure 2D) to bucain from *B. candidus *venom [55]; [56] and a neurotoxin homolog NTL4 [57]. Similar to bucain, this toxin may exhibit not-so-potent neurotoxicity.

The ESTs included 56 clones of non-conventional 3FTxs (fifth disulfide bridge in loop 1; [58]) (Figure 2E-G). Three clusters represented by BF648, BF402 and BF797 with 26, 4 and 2 clones respectively, which have few minor amino acid residue changes in the mature proteins. These transcripts encode proteins similar to candoxin with 80% identity (Figure 2E). Candoxin from *B. candidus *venom is known to bind reversibly to peripheral nAChRs and irreversibly to α7 nAChRs [59]. Functionally, these *B. flaviceps *proteins may exhibit similar, if not identical, properties. Second cluster BF11 (7 clones), BF661 (4 clones) and BF685 (single clone) shows 87% and 70% identity to two non-conventional toxins from *B. candidus *venom (Figure 2F). The other two isoforms are much closer (90% and 74% identity respectively). Similarly, BF533 (Singleton) showed 64% identity to both BM14 [60] and bulongin (R. M. Kini, unpublished data) from *B. multicinctus *and *B. candidus *venoms respectively (Figure 2G). BM14 binds to muscarinic M2 receptor subtypes with much lower affinities compared to typical muscarinic toxins from *Dendroaspis *venoms [60,61]. In BM 14, trinitrophenylation of the Lys residues (37th and 38th) abolished its binding to muscarinic acetylcholine receptor (mAChR). However, based on the lower structural similarity and the lack of the critical Lys residues (replaced by Ile and Pro), we speculate that the protein coded by BF533 may have distinct biological properties compared to BM14 and bulongin.

Interestingly, only one singleton, BF776, shows 100% match (except for three missing amino acid residues at the N-terminal) to κ-flavitoxin (long-chain neurotoxin 2) from the venom of *B. flaviceps flaviceps *[25,26] (Figure 2H). The observed truncation at the 5'end is likely to be due to its degradation of mRNA during total RNA extraction. κ- flavitoxin is a competitive neuronal acetylcholine receptor (nAChR) antagonist [25,26]. In general, κ- neurotoxins are highly specific antagonists of α3β2 and α4β2 receptors [62]. Interestingly, we did not find a transcript encoding α- bungarotoxin.

Due to the dominating role of α-, β- and κ- bungarotoxins in *Bungarus *species, the functional roles of other toxins have not been systematically explored. Of the eight major groups of 3FTxs in our *B. flaviceps *venom gland ESTs data, only κ- neurotoxins are fairly well characterized. Therefore, it will be interesting to study these toxins and identify the molecular targets.

During our sequencing studies, we observed a number of clones with part of their sequences missing. In translated sequence of BF229 and BF249 (both singletons), loop I and part of loop II was found to be missing. When BF229 was compared to the gene of the black-and-white spitting cobra (*Naja sputatrix*) encoding weak neurotoxin 10 (accession number AY081762) [63], we found that exon II was missing in this clone (data not shown). Thus these clones were probably generated by an error in splicing. Premature truncation was also observed in BF846 and BF600 (both singletons). In BF846, truncation is due to a dinucleotide deletion, whereas in BF600 the insertion of adenosine nucleotide has lead to a frame shift. It is not clear whether these aberrations are artefacts of cloning and sequencing or products of independent genes, this requires further investigation. In any case, these truncated transcripts constitute about 3% of 3FTxs and may not have any influence on the overall toxicity of the venom.

Recently, by the analysis of gene structures of viperid 3FTxs, we showed that some of these toxins evolve through the phenomenon of segment switch [13]. The analysis of cDNA sequences of *B. flaviceps *reveals that these toxins also appear to be evolving through accelerated segment switch in exons (Additional file 1). This phenomenon, named as ASSET, may be an alternative mechanism of accelerated evolution of snake venom toxins [13,64].

### β- bungarotoxin

β-bungarotoxin is one of the major lethal components found in the venom of *Bungarus *species. It targets the pre-synaptic terminal, where it causes the massive release of acetylcholine resulting in subsequent exhaustion of acetylcholine and inability to conduct an impulse and finally, paralysis. It is a heterodimeric covalent protein complex [16,17,65] (for a review see, [64]) composed of chain A similar to PLA_2 _and chain B similar to Kunitz-type SPI. It is a potent presynaptic neurotoxin [16,18]. We found three isoforms of chain A and four isoforms of chain B (for details, see below). Chain A transcripts represented 2.87% of all ESTs (13 ESTs, full length 11) and chain B transcripts 32.01% (145 ESTs, full length 96) (Figure 1B). This drastic difference in expression level is interesting, considering the fact that these two chains combine to an equimolar complex (β- bungarotoxin). This difference in expression levels requires further investigation. As the two chains belong to two different families, they will be discussed separately below.

### Kunitz-type Serine Protease Inhibitor (SPI) Family

Kunitz-type SPIs are one of the major groups of snake venom proteins mainly reported from the venoms of Elapidae and Viperidae snakes and they inhibit either trypsin or chymotrypsin [66-71]. Structurally they belong to the bovine pancreatic trypsin inhibitor (BPTI) family [72,73]. They have approximately 60 amino acid residues with six cysteine residues [74] arranged in a conserved sequence motif of C-8X-C-15X-C-4X-YGGC-12X-C-3X-C [75].

**Figure 3:**
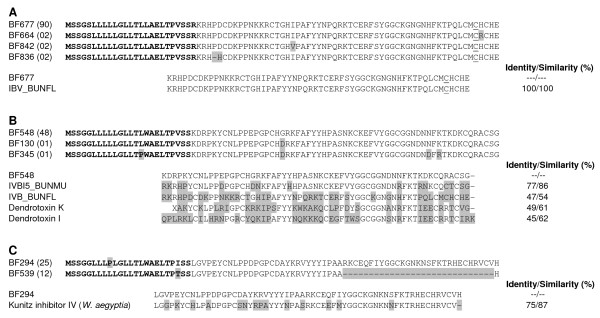
**Kunitz-type SPIs of *B. flaviceps *venom gland cDNA library**. Similar sequences were clustered together and a representative of the each cluster is presented. *B. flaviceps *Kunitz-type SPIs can be divided into three distinct groups (A, B and C). The deduced amino acid sequences of isoforms of each group are shown. The amino residues which are different from the representative of major isoform are shaded. The predicted signal peptide of the transcripts is shown in bold. The major isoforms from each group is aligned with closely related protein found in the database and the % identity and similarly are also shown in the figure. **A) **Putative clusters of β-bungarotoxin B chains of *B. flaviceps *and alignment with IVB_BUNFL (Q7T2Q6.1). The extra cysteine residue that is involved in the disulphide bridge with chain A is underlined. **B) **Putative Kunitz-type SPIs of *B. flaviceps *and alignment with IVBI5_BUNMU (Q1RPS9.1) chain B5 precursor of *Bungarus multicinctus*, IVB_BUNFL (Q7T2Q6.1) *B. flaviceps*, Dendrotoxin-K (P00981.2) and Dendrotoxin-I (P00979) from *Dendroaspis polylepis polylepis*. **C) **Putative Kunitz-type SPI and its truncated form. Alignment of the major isoform with Kunitz inhibitor IV (ABX82870.1) from *W. aegyptia *is also shown.

As mentioned above, the B chain of β- bungarotoxins is structurally similar to Kunitz-type SPI proteins. We found four clusters that exhibit high similarity to the B chain of β- bungarotoxins. Most common chain B isoform, BF677 (90 clones) is 100% identical to IBV_BUNFL, a chain B precursor isolated from *B. flaviceps *[15]. The other three isoforms (with two clones each), differ in amino acid residue from the major isoform in one or two positions (Figure 3A).

The second group of Kunitz-type SPI clones are similar to the B chain of β- bungarotoxins but lack the extra cysteine at the C-terminal end (Figure 3B). This extra cysteine residue is involved in interchain disulfide and is important for the complex formation of β-bungarotoxin (discussed above). The principal isoform BF548 with 48 clones shows similarity to chain B (B5) precursor of β- bungarotoxin from *B. multicinctus *[76]. Similarly BF130 and BF345 (both singletons) are also similar to chain B (B5) of β-bungarotoxin. Due to the absence of the extra cysteine, we believe that this polypeptide remains as monomer, similar to dendrotoxins [77] in the venom. Based on the high sequence identity and similarity to the chain B of β- bungarotoxins and dendrotoxins (Figure 3B), it is tempting to speculate that they might block voltage sensitive potassium channels [78,79]. This needs to be further investigated.

We found two clusters, BF294 and BF539 (25 and 12 clones) as the third group of Kunitz-type SPI clones. The major isoform is structurally similar to Kunitz inhibitor IV from *W. aegyptia *(Figure 3C). Interestingly, the minor isoform (represented by BF539) was found to be prematurely truncated. Comparison of mRNA sequence BF539 and a Kunitz-type SPI from the King Cobra (*Ophiophagus hannah*) (EU246693), and to the sequence of PILP-1 from *B. multicinctus *[74], reveals that 87 nucleotides from exon II is lost in these clones (Additional file 4). However the stop codon and 3'UTR encoded by Exon III is conserved in this clone. This splicing error could occur due to the presence of GT (splice start site) in exon II. The truncated mature protein has 28 amino acid residues with the first pair of cysteine residues. It would be interesting to study its biological properties.

**Figure 4:**
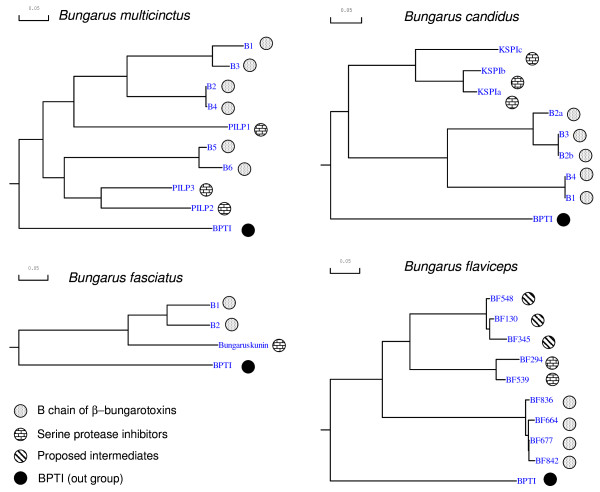
**Phylogenetic relationship of Kunitz-type SPI and β-bungarotoxin chain B of different *Bungarus *species**. The phylogenetic trees are constructed using BPTI as the out-group.

To understand the evolutionary relationships of Kunitz-type SPIs and B chains among the *Bungarus *species we constructed a phylogenetic tree using BPTI as an out group (Figure 4). The *B. flaviceps *precursors (BF677, BF664, BF842 and BF836) of B chains of β- bungarotoxins cluster separately from other two groups. The second group of transcripts (BF548, BF130 and BF345) clusters together with Kunitz-type SPI (BF294 and BF539) despite their similarities to chain B of β- bungarotoxins (Additional file 5). Thus most likely they are not the intermediates in the evolution of chain B. In the case of *B. candidus *and *B. fasciatus*, B chain cluster separately from the Kunitz-type SPIs. In *B. multicinctus *B chains, B1, B2, B3 and B4 cluster together and are closer to PILP-1, a SPI, whereas B chains B5 and B6 are closer to SPIs, PILP2 and PILP3.

### Phospholipase A2 (PLA_2_) Family

PLA_2 _enzymes are esterolytic enzymes which hydrolyze glycerophospholipids at the *sn*-2 position of the glycerol backbone, resulting in the release of lysophospholipids and free fatty acids. They are 116 to 124 amino acid residues long with an approximate molecular mass of 13-14 kDa. This family of enzymes have 12-14 conserved cysteine residues which form six or seven disulphide bridges [80]. Structurally, they share a similar protein folding pattern of a α-helical core, a backbone loop and a β- wing [81]. Functionally, they have a broad array of pharmacological effects which encompass neurotoxic, myotoxic, cardiotoxic, haemolytic, convulsive, anticoagulant, antiplatelet, oedema-inducing and tissue-damaging effects (For a review see [80,82]).

**Figure 5:**
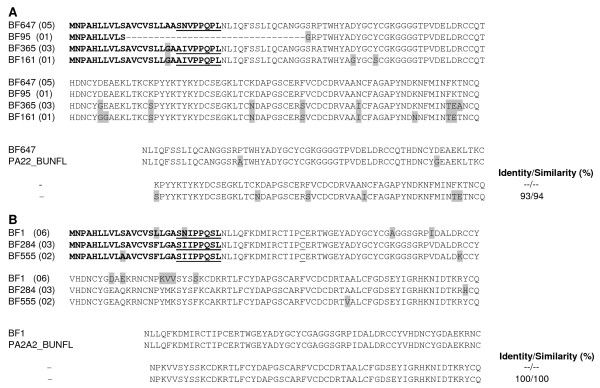
**Phospholipase A_2 _of *B. flaviceps *venom gland cDNA library**. Similar sequences were clustered together and a representative of the each cluster is presented. *B. flaviceps *PLA_2_s can be divided into two distinct groups (A and B). The deduced amino acid sequences of isoforms of each group are shown. The amino residues which are different from the representative major isoform are shaded. The predicted signal peptide of the transcripts is shown in bold and the propeptide is underlined. The major isoforms from each group is aligned with closely related protein found in the database and the % identity and similarly are also shown in the figure. **A) **Putative PLA_2 _precursors of *B*. *flaviceps *and alignment with PA22_BUNFL (Q7T2Q4.1) from *B. flaviceps*. **B) **Putative β-bungarotoxin chain A of *B. flaviceps *and alignment with PA2A2_BUNFL (Q7T1R1.1) from *B. flaviceps*. The extra cysteine residue involved in formation of disulphide bridge with chain A of β-bungarotoxin is underlined.

**Figure 6:**
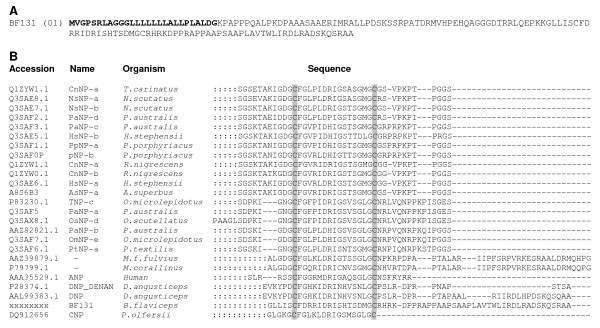
**Natriuretic peptide in *B. flaviceps *venom gland cDNA library**. **A) **The deduced amino acid sequence of the full length precursor is shown. The predicted signal peptide of the transcripts is shown in bold. The putative mature NP is boxed. **B) **Alignment of *B. flaviceps *NP with other snake venom NPs including human NP. The amino acid sequences were obtained from the database. The accession number, name of the protein and the organism are shown in the figure. The sequences were aligned manually and the gaps are filled with dashes, those amino acids which are not shown in the alignment are represented by ":". The cysteine residues involved in forming the 17 residue ring structure are highlighted in the figure.

Within the ESTs, 3.30% of the transcripts belong to PLA_2 _family (Figure 1B). Clones encoding PLA_2 _were grouped into two clusters (5 and 3 clones) and two singletons (Figure 5). Prediction of signal peptide using SignalP program indicates that these sequences have the eight amino acid propeptide sequences (underlined in the Figure 6) [83]. This propeptide sequence in BF647 is represented by "SNVPPQPL" whereas in BF365 and BF161 it is "AIVPPQPL" and there are two substitutions (SN is replaced with AI). Although similar propeptides are found in other elapid PLA_2 _enzymes, most mature proteins do not have them. Only a small number (group IB) retain them. The PLA_2 _isoforms are highly similar to each other except for few amino acid substitutions in the mature protein (Figure 5A). Clone BF95 has a deletion of 32 amino acid residues (9 residues from the signal peptide and 23 residues from the N-terminal end including the propeptide) as compared to the other PLA_2 _isoforms. Comparison of the BF95 sequence with the sea snake (*L. semfasciata*) PLA_2 _sequence reveals that there is a deletion of 99 bp from exon II (Additional file 6). Consequence of this deletion in exon II in its activity is not known and needs further investigation. In one of the clones (data not shown) the 5' end of the transcript was missing, this might be due to degradation of the mRNA during preparation. When compared to *B. flaviceps *PLA_2 _I (BAC77655) and PLA_2 _II (BAC77656) sequence [15], BF647, BF365 and BF161 show 93% identity (Figure 5A).

As mentioned above, the chain A of β- bungarotoxins is structurally similar to PLA_2 _enzymes. We found three clusters that exhibit high identity to chain A of β- bungarotoxins (Figure 5B). BF1 (6 clones) which has few amino acid residue changes both in the mature protein as well as in the signal peptide as compared to BF284 (3 clones) and BF555 (2 clones). But BF284 and BF555 have two residue differences; one amino acid substitution in the signal peptide and two others in the mature protein. Cluster BF1 is 100% identical to Chain A2 of β-bungarotoxin from *B. flaviceps *reported earlier whereas BF284 and BF555 show 97% identity to Chain A1 in the mature protein [15]. Multiple isoforms of Chain A of β- bungarotoxin has been reported from *Bungarus *species. Five isoforms (A1-A5) have been reported from a single species of *B. multicinctus *snake [84], but we have observed only three isoforms in *B. flaviceps *transcriptome.

### Natriuretic Peptide Family

**Figure 7:**
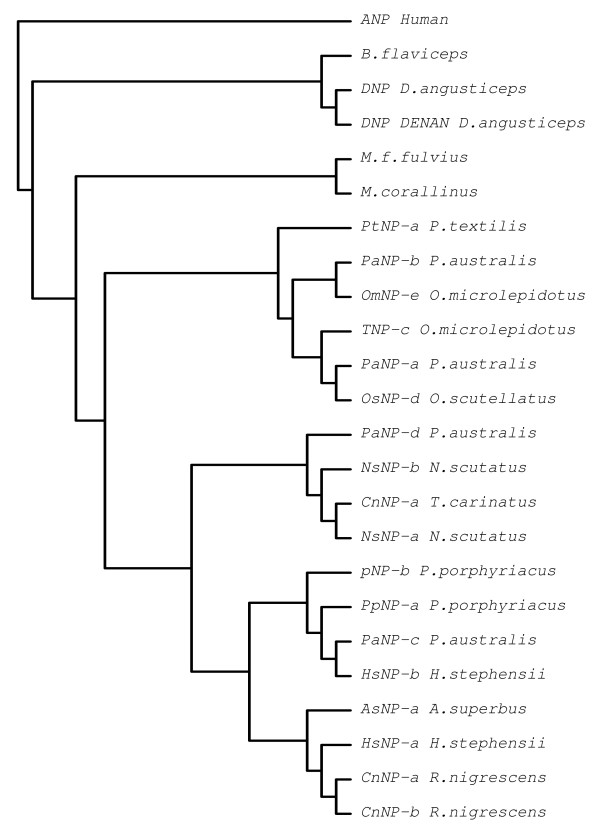
**Phylogenetic relationship of *B. flaviceps *NP with other snake venom NPs**. The phylogenetic tree was constructed using Human ANP as the out-group. The *B. flaviceps *NP cluster with Dendroaspis and Micururus species and remains separated from other Austalian elapids NPs.

Natriuretic peptides (NPs) are endogenous hormones initially found in mammals. Three mammalian NPs have been identified and characterized so far, which includes atrial NP (ANP), B-type NP (BNP) and C-type NP (CNP) [85-90]. In NPs a 17-residue ring structure, formed by an intra-molecular disulphide bond, is a highly conserved feature. Functionally, NPs are involved in various physiological processes such as regulation of water and electrolyte balance, cardiovascular system and cell growth [91-93]. Physiologically, in mammals NPs exhibit potent hypotensive and vasorelaxant properties and contributing to sodium and water retention [90,94-96]. The first NP of snake venom was isolated from *Dendroaspis angusticeps *venom and is known as *Dendroaspis *natriuretic peptide (DNP) [97]. It lowers the blood pressure through vasodilation [98]. Interestingly two clones (0.44%) encoding natriuretic peptides were identified in *B. flaviceps *library; one of them was partial while the other (BF131) was a full length transcript (Figure 6A). This is the first full length mRNA sequence of an elapid NP precursor. When compared to CNP precursors of crotalid snakes, *B. flaviceps *NP precursor lacks the BPP domain and part of the linker sequence (data not shown) similar to colubrid NP precursor reported from the Duvernoy's (venom) gland transcriptome of *Philodryas olfersii *[99]. However, *P. olfersii *encodes for CNP type molecule, whereas *B. flaviceps *NP belongs to the ANP/BNP family due to the C-terminal extension (Figure 6B). This is the first report of NPs in any Asian elapid. We constructed a phylogenetic tree to understand the evolutionary relationships of ANP/BNP found in different snakes. *B. flavipceps *ANP/BNP was found to be closely related to African mamba (*Dendroaspis*) and coral snake (*Micrurus*) NPs, but distantly related to the Australian elapid counterparts (Figure 7). However the presence of these transcripts in the protein level need to confirmed through proteomic approaches.

### Other families

CRISPs (2 clones), and C-type lectins (2 clones) transcripts were found, in a level of 0.44% of the total ESTs (Additional file 7). The CRISPs transcripts showed high similarity to latisemin reported from *Laticauda semifasciata*, oharin precursor from *Ophiophagus hannah*, pseudechetoxin-like protein precursor from *Oxyuranus scutellatus and to *kaouthin-2 precursor from *Naja kaouthia *[100-102]. Latisemin, ophanin and tigrin block potassium-stimulated smooth muscle contraction [102]. In CRISPs N-terminal pathogenesis-related protein-1 (PR-1) domain and the C-terminal cysteine-rich domain are conserved. PR-1 is known to be important for recognition of the target molecule and this domain. C-type lectins are non-enzymatic proteins which interact with carbohydrate moieties in the presence of Ca^2+ ^ions and usually possess the highly conserved carbohydrate recognition domain (CRD) [103,104]. One full length and other partial C-type lectins were obtained in this library. The full length transcripts have seven cysteine residues hence they are likely to exist as covalent homodimers in the venom [105]. Analysis of the translated sequence reveals a Ca^2+ ^binding site [105,106], suggesting that it is a Ca^2+^-dependent C-type lectin.

### Cellular transcripts

The cellular transcripts (125 EST) constitute about 20.56% of the total transcripts. Some of the important house-keeping ESTs include: ribosomal protein (24 clones), protein Sec61 beta subunit (4 clones) involved in protein transport, eukaryotic translation initiation factor (3 clones) and eukaryotic translation elongation factor 1 beta 2 (3 clones), which are involved in protein transcription and translation. The proteins encoded by these transcripts are involved in protein synthesis and secretion. Other house-keeping transcripts involved in cellular functions were: NADH dehydrogenase (3 clones); ubiquitin C (2 clones); ADP-ribosylation factor-like (2 clones); Cytoplasmic actin type 5; ubiquinol-cytochrome c reductase; sodium-dependent dicarboxylate transporter; Succinate-CoA ligase; Type 1 glutamine amidotransferase; L-lactate dehydrogenase; and a DNA bindng protein (zinc finger) (1 clone each).

## Conclusion

Analysis of 845 ESTs of the venom gland of the Red-headed krait (*B. flaviceps*) shows that 3FTxs and B chains of β-bungarotoxins are the main components of the venom. Most of the 3FTxs transcripts described here are structurally different from the previously characterized 3FTxs and hence they are likely to show distinct biological activities. We found a group of Kunitz-type SPIs highly similar to B chain of the β-bungarotoxin, but without the extra cysteine involved in interchain disulfide. Identification of ANP/BNP underscores the importance of transcriptome analysis in identifying low abundant proteins. Thus our study provides a platform to initiate the characterization of several novel proteins found in *B. flaviceps *venom gland.

## Methods

### Collection of venom glands and liver

A juvenile specimen of *Bungarus flaviceps *(Malaysia) was collected in Malaysia and imported to Europe. Venom was first obtained manually by sliding pipette tips over each fang, a technique standard for milking smaller elapid snakes. Venom was lyophilized and stored at -80°C. Four days later, when mRNA production is assumed to be maximal [107], the snake was anesthetized with Zoletil (Zolazepam and Tiletamine) and sacrificed by decapitation. The venom glands and liver were carefully but quickly dissected, cut into small pieces of 2-3 mm, and stored in RNAlater solution (Ambion, City, Country) in a ten-fold ratio to the volume of the glands, and stored at -80°C up until use.

### Construction of cDNA library and DNA sequencing

Total RNA was extracted from both the venom glands using the RNeasy^® ^Mini kit from Qiagen (Valencia, CA, USA). The quality of the RNA was tested using gel electrophoresis. Using Creator™ SMART™ cDNA library construction kit from Clontech Laboratories (Palo Alto, CA, USA), the following was achieved: (i) synthesis of first strand cDNAs from the total RNAs; (ii) synthesis of double-stranded cDNAs from the first strand cDNAs; and (iii) purification of double-stranded cDNAs using the CHROMA SPIN-400 column. Purified double-stranded cDNAs were cloned into the pCR^®^2.1-TOPO^® ^vector from Invitrogen (Carlsbad, CA, USA). Recombinant TOPO vector was transformed into DH5α competent cells. After plating onto ampicillin/IPTG/X-gal Luria Broth (LB) agar plates, the transformed DH5α cells were subjected to blue/white screening. Individual white colonies were randomly selected and grown in LB added with ampicillin. Plasmids were then purified using the GeneAll^® ^Exprep™ Plasmid Quick kit from GeneAll Biotechnology Co., Ltd (Songpa-gu, Seoul, Korea). The presence and size of inserts in the plasmids were confirmed using EcoRI digestion. Plasmids containing inserts larger than 200 bp were selected for DNA sequencing. The plasmids were sequenced using the chain termination method [108] using the ABI PRISM^® ^BigDye Terminator v3.1 Cycle Sequencing kit and ABI PRISM^® ^3100 automated DNA sequencer from Applied Biosystem (Foster City, CA, USA). Representative full length cDNA sequences from each cluster or singleton were submitted to NCBI database. The accession numbers for 3FTx families were from GU190789 to GU190804, Kunitz-type SPI GU190805 to GU190810 β-bungarotoxin B Chain GU1908011 to GU190814, PLA_2 _GU190815 to GU190817, β-bungarotoxin B Chain GU190818 to GU190820, Natriuretic peptide GU190821 and C-type lectin GU190822.

### Bioinformatics analyses

Vector and adaptor sequences were removed from the DNA sequences before translating them in all three frames for identification of the open reading frame (ORF). The trimmed DNA sequences and their correct ORF protein sequences were queried against nucleotide and protein databases using NCBI BLASTn and BLASTp http://blast.ncbi.nlm.nih.gov/Blast.cgi respectively to predict their putative functions. SignalP 3.0 server http://www.cbs.dtu.dk/services/SignalP/ was used for the prediction of signal peptide of the translated sequences. The similarity of transcripts to known toxin sequences available in the database was used as the first criterion for cataloguing transcripts of putative toxins. If the transcripts are novel with an ORF and do not show any similarity with any sequences of the database then we have used two criteria to identify potential novel toxins. The protein product should have a signal peptide (as venom proteins are secretory proteins) and rich in Cys residues (most venom toxins are rich in Cys residues). Multiple sequence alignments (MSA) were carried out using either Clustalx or DNAMAN version 4.15 (Lynnon Corporation, Pointe-Claire, Quebec, Canada; http://www.ebi.ac.uk/Tools/clustalw2/) or manually. Protein sequences were obtained from the NCBI database and sequence alignments were done using Clustalx. Phylogenetic trees were generated using Clustalx which employs Bootstrap N-J tree method and the tree was viewed using tree viewer.

## Authors' contributions

ASS and RD worked on the project and drafted the manuscript. RD analyzed the resulted and interpreted. FJV supplied the tissue samples and interpreted the results. RMK is the principal investigator who designed the experiment, analyzed the data and critically reviewed the manuscript. All the authors have approved the final version of the manuscript.

## Supplementary Material

Additional file 1 **Transcripts submitted to database showing similarity to snake venom protein family.** Numbers of transcripts, accession number, search programme and E values are shown in the table. Click here for file

Additional file 2 **% of venom proteins families observed in venom gland transcriptome.** Comparison of different toxin families observed in transcriptome of elapid and viperid venom gland. Click here for file

Additional file 3 **ASSET in 3FTxs of B. flaviceps.** Alignment of 3FTx of B. flaviceps showing Accelerated Segment Switch in Exon to alter Targeting (ASSET). The segments which are similar are shown in same color where as segments which are dissimilar are shown in different color. The clone name and the number of clones are also shown in the figure. Click here for file

Additional file 4 **Premature truncated kunitz type SPI from *B. flaviceps***. Comparison of protein and nucleotide of truncated kunitz type SPI from *B. flaviceps *with EU246693 from *Ophiophagus hannah*. Exons are highlighted with different colors, Exon I is highlighted with red color, Exon II with Blue and Exon III is in grey color. 87 Nucleotides are deleted from the exon II of BF539 as shown with dashes in the figure. Comparison of the mRNA sequence of BF539 with BF294 reveals that a dinucleotide "GT" (underlined and highlighted in red letter) is present at the end of the exon II of BF539. The splicing error could be due to change in this base substitution. However the exon III is intact as stop codon and one of the amino acid residue is encoded by the exon III. Click here for file

Additional file 5 **Phylogenetic relationship of B chain and Kunitz SPI of different *Bungarus *species**. Kunitz type SPI and B chain of β-bungarotoxin of Bungarus sp was obtained from the database and phylogenetic tree was constructed to understand the relationship between kunitz SPI and B chain of β-bungarotoxin. Click here for file

Additional file 6 **Comparison of BF95 from *B. flaviceps *with *Laticauda semifasciata *PLA_2_(AB062439)**. Exons are highlighted with different colors, Exon I is highlighted in green color; Exon II in magenta; Exon III in dark blue and Exon IV in grey. In BF95, part of the exon II (99 bp) is missing as shown in the figure with dashes. Click here for file

Additional file 7 **CRISPs and C-type lectins**. **A) **Transcripts encoding CRISPs (BF53), found in this venom gland cDNA library. **B) **C-type lectins (BF53) found in this venom gland cDNA library. One of the C-type lectin found in this venom gland cDNA library was truncated in the 5' end (BF764). The amino acid residues of C-type lectin involved in binding to the Ca^2+ ^are highlighted with green color and the cysteine residues are highlighted. Click here for file
