# Supplementary material for: Transcriptomic analysis of the venom gland of the red-headed krait (Bungarus flaviceps) using expressed sequence tags
Source: BMC Mol Biol. 2010 Mar 29;11:24. doi: 10.1186/1471-2199-11-24 (PMC2861064; doi:10.1186/1471-2199-11-24)
Supplement: Additional file 1 — Transcripts submitted to database showing similarity to snake venom protein family. Numbers of transcripts, accession number, search programme and E values are shown in the table. [file 1471-2199-11-24-S1.PDF]

Transcript	Nos	Acc. No	Search	Similarity	E Value	Toxin family
BF601	46	GU190789	BLASTp	Candiduxin-1 (*Bangurus candidus*)	1e-21	3FTx
BF421	02	GU190790	BLASTp	Candiduxin-1 (*Bangurus candidus*)	1e-21	3FTx
BF141	01	GU190791	BLASTp	Candiduxin-1 (*Bangurus candidus*)	1e-15	3FTx
BF478	42	GU190792	BLASTp	Three finger toxin III (*Walterinnesia aegyptia*)	6e-16	3FTx
BF296	02	GU190793	BLASTp	Three finger toxin III (*Walterinnesia aegyptia*)	7e-16	3FTx
BF9	13	GU190794	BLASTp	Neurotoxin-like protein (*Bungarus multicinctus*)	2e-28	3FTx
BF222	07	GU190795	BLASTp	Neurotoxin-like protein (*Bungarus multicinctus*)	4e-27	3FTx
BF648	22	GU190796	BLASTp	Long neurotoxin homolog (*Bungarus multicinctus*)	4e-27	3FTx
BF402	06	GU190797	BLASTp	Long neurotoxin homolog (*Bungarus multicinctus*)	3e-29	3FTx
BF797	02	GU190799	BLASTp	Long neurotoxin homolog (*Bungarus multicinctus*)	1e-28	3FTx
BF11	07	GU190800	BLASTp	Neurotoxin Ntx4 (*Bungarus candidus*)	1e-32	3FTx
BF661	04	GU190801	BLASTp	Neurotoxin Ntx4 (*Bungarus candidus*)	3e-34	3FTx
BF685	01	GU190802	BLASTp	Neurotoxin Ntx4 (*Bungarus candidus*)	1e-34	3FTx
BF776	01	GU190803	BLASTp	Kappa-flavitoxin (*Bungarus flaviceps flaviceps*)	5e-29	3FTx
BF533	01	GU190804	BLASTp	cardiotoxin-like protein [*Bungarus multicinctus multicinctus*]	7e-25	3FTx
BF294	25	GU190806	BLASTp	Kunitz inhibitor IV (*Walterinnesia aegyptia*)	1e-25	SPI
BF539	12	GU190807	BLASTp	Kunitz inhibitor IV (*Walterinnesia aegyptia*)	7e-08	SPI
BF548	48	GU190808	BLASTp	Beta-bungarotoxin b5 chain precursor (*Bungarus multicinctus)*	6e-26	SPI
BF130	01	GU190809	BLASTp	Beta-bungarotoxin b5 chain precursor (*Bungarus multicinctus)*	1e-26	SPI
BF345	01	GU190810	BLASTp	Beta-bungarotoxin b5 chain precursor (*Bungarus multicinctus)*	1e-25	SPI
BF677	90	GU190811	BLASTp	Beta-bungarotoxin B chain precursor (*Bungarus flaviceps*)	9e-42	SPI
BF664	02	GU190812	BLASTp	Beta-bungarotoxin B chain precursor (*Bungarus flaviceps*)	5e-41	SPI
BF842	02	GU190813	BLASTp	Beta-bungarotoxin B chain precursor (*Bungarus flaviceps*)	1e-41	SPI
BF836	02	GU190814	BLASTp	Beta-bungarotoxin B chain precursor (*Bungarus flaviceps*)	2e-30	SPI
BF365	03	GU190815	BLASTp	Phospholipase A2II precursor (*Bungarus flaviceps*)	4e-81	PLA_2
BF161	01	GU190816	BLASTp	Phospholipase A2II precursor (*Bungarus flaviceps*)	9e-72	PLA_2
BF647	05	GU190817	BLASTp	Phospholipase A2II precursor (*Bungarus flaviceps*)	3e-66	PLA_2
BF284	03	GU190818	BLASTp	Beta-bungarotoxin A1 chain precursor (*Bungarus flaviceps*)	9e-81	PLA_2
BF555	02	GU190819	BLASTp	Beta-bungarotoxin A1 chain precursor (*Bungarus flaviceps*)	2e-71	PLA_2
BF1	06	GU190820	BLASTp	Beta-bungarotoxin A1 chain precursor (*Bungarus flaviceps*)	1e-80	PLA_2
BF131	01	GU190821	BLASTp	Natriuretic peptide (*Micrurus corallinus)*	1e-07	NP
BF527	02	GU190822	BLASTp	C-type lectin mannose binding isoform 4 (*Oxyuranus scutellatus*)	8e-75	C-type lectin
