# Supplementary material for: Transcriptomic analysis of the venom gland of the red-headed krait (Bungarus flaviceps) using expressed sequence tags
Source: BMC Mol Biol. 2010 Mar 29;11:24. doi: 10.1186/1471-2199-11-24 (PMC2861064; doi:10.1186/1471-2199-11-24)
Supplement: Additional file 2 — % of venom proteins families observed in venom gland transcriptome. Comparison of different toxin families observed in transcriptome of elapid and viperid venom gland. [file 1471-2199-11-24-S2.PDF]

**Family**	**Snake**	**3FTx (%)**	**PLA 2 (%)**	**Serine Proteases (%)**	**Mettalo protease (%)**	**C-type lectin**	**C-type lectin like**	**CRISPs**	**Reference**
Elapidae	*Micrurus corallines*	24	15	0.07	0.3	4.8	-	-	Leao et al., 2009
	Bungurus flaviceps	39.29	6.18	-	-	0.44	-	0.44	This study
	Austrelaps. labialis	45	33	-	3	-	2	8	Doley et al., 2008
	Lapemis curtus	43.3	9.8	-	-	-	-	2.2	Pahari et al., 2007
	Acalyptophis peronii	63.7	5.2	-	-	-	-	-	Pahari et al., 2007
Viperidae	*Akistrodon acutus*	-	0.7	1.3	79.9	13	-	-	Liu Qinghua et al., 2006
	Sistrurus catenatus edwardsii	0.83	28.06	37.5	12.22	1.4	-	6.6	Pahari et al., 2007
	Bothrops insularis	-	6.7	9.6	41.7	14.5	-	0.6	Ina´cio de L.M. et al., 2002
	Bitis gabonica	-	5.0	13	30	-	6.7	-	Francischetti et al., 2004
	Bothrops jararaca	-	0.7	28.5	53.1	8.3	-	1.6	Cidade et al., 2006
	Bothrops atrox	-	4.6	2.8	21.3	2.3	-	0.2	Neiva et al., 2009
