# Supplementary material for: Transcriptomic analysis of the venom gland of the red-headed krait (Bungarus flaviceps) using expressed sequence tags
Source: BMC Mol Biol. 2010 Mar 29;11:24. doi: 10.1186/1471-2199-11-24 (PMC2861064; doi:10.1186/1471-2199-11-24)
Supplement: Additional file 3 — ASSET in 3FTxs of B. flaviceps. Alignment of 3FTx of B. flaviceps showing Accelerated Segment Switch in Exon to alter Targeting (ASSET). The segments which are similar are shown in same color where as segments which are dissimilar are shown in different color. The clone name and the number of clones are also shown in the figure. [file 1471-2199-11-24-S3.PDF]

```
BF601 (46) NVCYTHESANPKTS--VLCGYGTIFCYKSSWIYRGVEKIE-RGCASACPDMKPNGKYIYCCTRDECND
BF421 (02) NVCYTHESANPKTS--VLCGYGTIFCYKSSWIYRGVEKIE-RGCASACPDMKPNGKYIYCCTRDECND
BF141 (01) NVCYTHESANPKTS--VLCGYGTIFCYKSSWIYRGVEKIE-RGCASACPDMKPNGKHIYCCTRDECND
BF9   (13) KLCYNHQSTNPKTT--ELCGHSMYFCYKNSWIYRGVEKIE-RGCSLTCPDIKSNGKYIYCCTRDKCND
BF648 (26) IKCKICQFNTCRPGELKVCSGEEIYCFKESWSTARGTRIE-RGCTATCPKGSVYGNYVLCCTTDECNI
BF402 (06) IKCKICQFNTCRPGELKVCSGEEIYCFKESWSTARGTRIE-RGCTATCPKGSVYGNYVLCCTTDECNI
BF797 (02) IKCKICQFNTCRPGELKVCSGEEIYCFKESWSTARGTRIE-RGCTATCPKGSVYGNYVLCCTIDECNI
BF748 (42) RICYNQQSTTPPTT--ENCEPGKNVCYKMYFSDHRGTRSS-RGCVATCPTNNRYDR-VVCCEKDKCNM
BF296 (02) RICYNQQSTTPPTT--ENCEPGKNVCYKMYFSDHRGTRSS-RGCVVTCPTNNRYDR-VVCCEKDKCNM
BF11  (07) LTCLICPEKYCQKV--HTCQDAEKICFKRFYEGKQLGKKFPRGCAATCPEAKPHE-IVECCSTDKCNK
BF661 (04) LTCLICPEKYCQKV--HTCRDGEKICFKRFYEGKRFGKKFPRGCAATCPEAKPHE-IVECCSTDKCNK
BF685 (01) LTCLICPEKYCQKV--HTCRDGEKICFKRFYEGKRFGKKFPRGCAATCPEAKPHE-IVECCSTDKCNK
BF222 (07) RKCLTKYSRDNESS--KTCPSGQNVCFKKWEMGNSSEKNAKRGCIAACPKPKKNEM-IQCCSKDKCNK
```
