# Supplementary material for: Transcriptomic analysis of the venom gland of the red-headed krait (Bungarus flaviceps) using expressed sequence tags
Source: BMC Mol Biol. 2010 Mar 29;11:24. doi: 10.1186/1471-2199-11-24 (PMC2861064; doi:10.1186/1471-2199-11-24)
Supplement: Additional file 4 — Premature truncated kunitz type SPI from B. flaviceps. Comparison of protein and nucleotide of truncated kunitz type SPI from B. flaviceps with EU246693 from Ophiophagus hannah. Exons are highlighted with different colors, Exon I is highlighted with red color, Exon II with Blue and Exon III is in grey color. 87 Nucleotides are deleted from the exon II of BF539 as shown with dashes in the figure. Comparison of the mRNA sequence of BF539 with BF294 reveals that a dinucleotide "GT" (underlined and highlighted in red letter) is present at the end of the exon II of BF539. The splicing error could be due to change in this base substitution. However the exon III is intact as stop codon and one of the amino acid residue is encoded by the exon III. [file 1471-2199-11-24-S4.PDF]

A

Protein sequences

```
EU246693     MSSGRLLLLLGLLTLWAELTPVSGLGRPKFCELPPEPGLCNARKTFFYYSLHSHACQKFIYGGCGGNANKFKTIDECHRTCVG-&
BF539        MSSGGLLLLLGLLTLWAELTPTSSLGVPEYCNLPPDPGPCDAYKRVYYYIPAA------------------------------H&
BF294 (25)   MSSGGLLLPLGLLTLWAELTPISSLGVPEYCNLPPDPGPCDAYKRVYYYIPAARKCEQFIYGGCKGNKNSFKTRHECHRVCV-H&
```

B

Nucleotide sequence (mRNA)

```
EU246693     ATGTCTTCTGGACGTCTTCTTCTCCTGCTGGGACTCCTCACCCTCTGGGCAGAGCTGACCCCCGTCTCCGGCCTGGGCCGTCCAAAGTTCTGTGAACTGCCTCCTG
BF539        ATGTCTTCTGGAGGTCTTCTTCTCCTGCTGGGACTCCTTACCCTCTGGGCGGAGCTGACCCCCATCTCCAGCCTGGGAGTTCCAGAGTATTGTAATCTGCCTCCTG
BF294        ATGTCTTCTGGAGGTCTTCTTCTCCTGCTGGGACTCCTTACCCTCTGGGCGGAGCTGACCCCCATCTCCAGCCTGGGAGTTCCAGAGTATTGTAATCTGCCTCCTG

EU246693     AACCCGGATTATGCAACGCCCGTAAAACTTTCTTCTACTACAGCCTGCATTCACATGCATGCCAAAAGTTTATTTATGGTGGCTGTGGGGGCAATGCCAACAAATT
BF539        ACCCCGGACCATGTGATGCCTATAAACGTGTCTACTACTACATCCCGGCTGCAC----------------------------------------------------
BF294        ACCCCGGACCATGTGATGCCTATAAACGTGTCTACTACTACATCCCGGCTGCACGTAAATGCGAACAGTTTATTTATGGTGGATGCAAAGGGAATAAGAATAATTT

EU246693     TAAGACCATAGATGAATGCCACCGCACCTGTGTTGGATGACCAATGAGGAGACCCACCCAGAATGGATCCAGTGTTCCAACTTGACCCAAAGACCCTGCTTCTGCC
BF539        -----------------------------------ATTGACCAATGAGGAGACCCTCCCAGAATGGATCCAGTGTTCCAACTTGACCCAAAGAACCTGCTTCTGCC
BF294        TAAGACCAGACATGAATGCCACCGCGTCTGTGTTCATTGACCAATGAGGAGACCCTCCCAGAATGGATCCAGTGTTCCAACTTGACCCAAAGAACCTGCTTCTGCC

EU246693     CTGGACCACCTTGAGACCCTCCCCCCAAACCCCACCCTGGGCTCACTCCTTTTTCTCTGCAATAAAGCTTTGGTTCCAGCTGCTAAAAAAAAAAAAAAAAA
BF539        CTGGACCACCCTGGAGACCCTCCCCCAGACCCCACCCTGGGCTCATTCCTTTTTCTCTGCAATAAAGCTTTGGTTCCAGCTGCAAAAAAAAAAAAAAAAAA
BF294        CTGGACCACCCTGGAGACCCTCCCCCAGACCCCACCCTGGGCTCATTCCTTTTTCTCTGCAATAAAGCTTTGGTTCCAGCTGCAAAAAAAAAAAAAAA
```
