# Supplementary figures and images for: Transcriptomic analysis of the venom gland of the red-headed krait (Bungarus flaviceps) using expressed sequence tags
Source: BMC Mol Biol. 2010 Mar 29;11:24. doi: 10.1186/1471-2199-11-24 (PMC2861064; doi:10.1186/1471-2199-11-24)

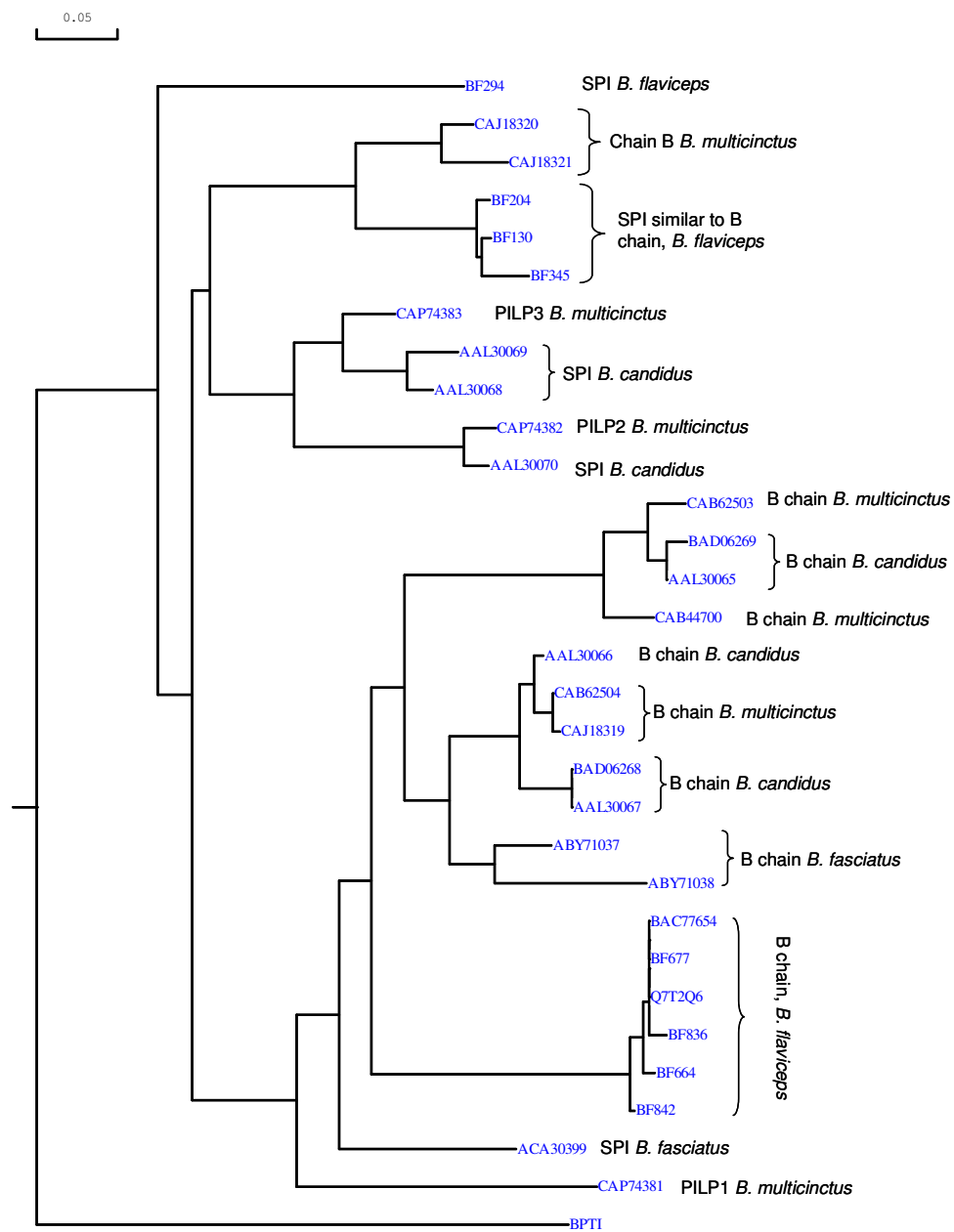

Supplement: Additional file 5 — Phylogenetic relationship of B chain and Kunitz SPI of different Bungarus species. Kunitz type SPI and B chain of β-bungarotoxin of Bungarus sp was obtained from the database and phylogenetic tree was constructed to understand the relationship between kunitz SPI and B chain of β-bungarotoxin. [file 1471-2199-11-24-S5.PDF]
