# Supplementary material for: Transcriptomic analysis of the venom gland of the red-headed krait (Bungarus flaviceps) using expressed sequence tags
Source: BMC Mol Biol. 2010 Mar 29;11:24. doi: 10.1186/1471-2199-11-24 (PMC2861064; doi:10.1186/1471-2199-11-24)
Supplement: Additional file 6 — Comparison of BF95 from B. flaviceps with Laticauda semifasciata PLA2(AB062439). Exons are highlighted with different colors, Exon I is highlighted in green color; Exon II in magenta; Exon III in dark blue and Exon IV in grey. In BF95, part of the exon II (99 bp) is missing as shown in the figure with dashes. [file 1471-2199-11-24-S6.PDF]

A

Protein Sequence

```
BF95       MNPAHLLVLS--------------------------------GRPTWHYADYGCYCGKGGGGTPVDELDRCCQTHDNCYDEAEKLTKCKPYYKTYKYDCSEG
AB062439   MYPAHLLVLLAVCVSLLGASAIPPLPLNLVQFTYLIQCANKGSRPSYHYADYGCYCGAGGSGTPVDELDRCCKIHDDCYGEAEK-MGCYPKLTMYNYYCGTE

BF95       KLTCKDAPGSCERFVCDCDRVAANCFAGAPYNDKNFMINFKTNCQ
AB062439   GPYCNTKTD-CQRYVCACDLKAAKCFARSPYNNKNYNIDTSKRCK
```

B

Nucleotide sequence (mRNA)

```
BF95       ATGAATCCTGCTCACCTTCTGGTCCTGTCGGC---------------------------------------------------------------------
AB062439   ATGTATCCTGCTCACCTTCTGGTCCTGTTGGCAGTTTGTGTCTCCCTCTTAGGAGCCTCCGCCATTCCTCCCCTGCCTCTCAACCTCGTATACAATTCACC

BF95       -------------------------------AGACCTACTTGGCATTATGCGGACTACGGTTGCTACTGTGGCAAAGGAGGTGGCGGACACCAGTAGACGA
AB062439   TACTTGATTCAATGTGCCAACAAAGGCAGCAGACCTTCTTATCATTATGCGGACTACGGATGCTACTGTGGCGCAGGAGGTAGCGGAACACCGGTAGACGA

BF95       ATTGGATAGGTGCTGCCAGACTCATGACAACTGCTATGATGAAGCCGAAAAACTTACCAAATGCAAGCCCTACTACAAGACATATAAATACGACTGTTCCG
AB062439   GTTGGATAGGTGCTGCAAAATACATGATGACTGCTATGGTGAAGCCGAAAAAATGGG---ATGCTACCCAAAGTTGACGATGTATAATTACTACTGTGGCA

BF95       AAGGCAAACTCACCTGCAAAGATGCCCCAGGCAGCTGTGAACGTTTTGTCTGTGATTGTGACCGCGTAGCAGCCAACTGCTTCGCCGGAGCTCCTTACAAC
AB062439   CAGAGGGACCCTACTGCAATACAAAAACGGAT---TGTCAACGTTATGTGTGTGCTTGTGACCTCAAGGCAGCCAAATGCTTTGCCAGATCCCCTTACAAC

BF95       GACAAGAACTTCATGATTAACTTCAAGACAAATTGTCAATGA
AB062439   AACAAGAACTACAATATCGACACCAGCAAACGTTGCAAATGA
```
