# Supplementary material for: Transcriptomic analysis of the venom gland of the red-headed krait (Bungarus flaviceps) using expressed sequence tags
Source: BMC Mol Biol. 2010 Mar 29;11:24. doi: 10.1186/1471-2199-11-24 (PMC2861064; doi:10.1186/1471-2199-11-24)
Supplement: Additional file 7 — CRISPs and C-type lectins. A) Transcripts encoding CRISPs (BF53), found in this venom gland cDNA library. B) C-type lectins (BF53) found in this venom gland cDNA library. One of the C-type lectin found in this venom gland cDNA library was truncated in the 5' end (BF764). The amino acid residues of C-type lectin involved in binding to the Ca2+ are highlighted with green color and the cysteine residues are highlighted. [file 1471-2199-11-24-S7.PDF]

A

BF53 (02)

MIAFIVLLSLAAVLQPSSGIVDFASESSNKRENQKQIVDKHNALRRSVKPTARNMLKMEWNSNAAQNAKRWADRCTFVHSPSHLRTVGKFSCGENLFMSSQPYAWSKVIQSWYDEYK
NFVYGVGANPPGSVIGHFTQIVWYKSHLLGCAAAKCSSSKYIYVCQYCPAGNIIVSIATPYKSGPSCGDCPSACDNGLCTNPCKHNDVFLNCKSLAKQSRCQTEWIKSKCAAS
CFCQTEII

B

```
BF527  MERFIFLSLGLLVVALSLRGTGADDPQCPFDWSFYNGHCYKVFKKLKNWRDAEMSCRQQEEGSHLASIQSWAESAYVANLISHNVLLTNVWIGLSDPWKQRIWYWSDGSRFRYK
BF764  ------------------------------------------------------SCRQQEEGSHLASIQSWAESAYVANLISHNVLLTNVWIGLSDPWKQRIWYWSDGSRFRYK

BF527  SWKLGEPNNFLWNEYCVELWSLSGYLGWNDQNCGFRRYCVCKFQPQGEGSTW
BF764  SWKLGEPNNFLWNEYCVELWSLSGYLGWNDQNCGFRRYCVCKFQPQGEGSTW
```
